# An unusual pathology of a nasal tip mass in a 28-year-old female

**DOI:** 10.1016/j.radcr.2024.12.061

**Published:** 2025-01-23

**Authors:** Shahin Rajaeih, Farshad Riahi, Behnaz Bouzari, Sam Mirfendereski

**Affiliations:** a ENT and Head and Neck Research Center and Department, The Five Senses Health Institute, Firoozgar Hospital, Iran University of medical Sciences, Tehran, Iran; b Department of Radiology, Isfahan University of Medical Sciences, Isfahan, Iran; c Assistant Professor of Pathology, Department of pathology, Iran University of Medical Sciences, Tehran, Iran; d Assistant Professor of Radiology, Department of Radiology, Isfahan University of Medical Sciences, Isfahan, Iran

**Keywords:** Nasal tip mass, Keratinous cyst, Apex nasi

## Abstract

The nose is a frequently affected site for skin cancers, thus it should always be considered when addressing pathologies of the external apex nasi. This case report presents a 28-year-old woman with an atypical nasal tip mass, characterized as a keratinous cyst demonstrating a multinucleated giant cell reaction and calcification. The mass, which had gradually enlarged over 3 years, was successfully removed using an open rhinoplasty technique. Preoperative imaging studies, including ultrasound, noncontrast CT, and MRI, revealed specific characteristics of the mass, aiding in diagnosis and surgical planning. The study underscores the need for increased awareness about nasal tip deformities caused by keratinous cysts, emphasizing the need for comprehensive diagnostic and therapeutic strategies to improve patient outcomes.

## Introduction

The apex nasi is a complicated area with two components: the soft tissue and cartilaginous structure of the anterior nasal region and the anterior nasal cavity. Comprehending local anatomy is essential for recognizing pathology and patterns of disease dissemination. Both CT and MR imaging can tell us a lot about this subject. CT images show bone structures, while MR images are more specific because they show more contrast between soft tissues [1].

Skin cancers that happen often usually affect the tip of the nose. However, imaging pitfalls like perineural tumor dissemination or rhinophyma can make things harder. Some uncommon growth or noncancerous problems, like nasolabial cysts and pyriform aperture stenosis, are only found in this area. Vascular lesions, nasal septal hematoma, and abscess may also affect the apex nasi [2].

Here we reported a 28-year-old female with a lesion on the tip of her nose that was diagnosed as a keratinous cyst, along with a multinucleated giant cell reaction and calcification which is a rare condition.

## Case presentation

**Fig. 1:**
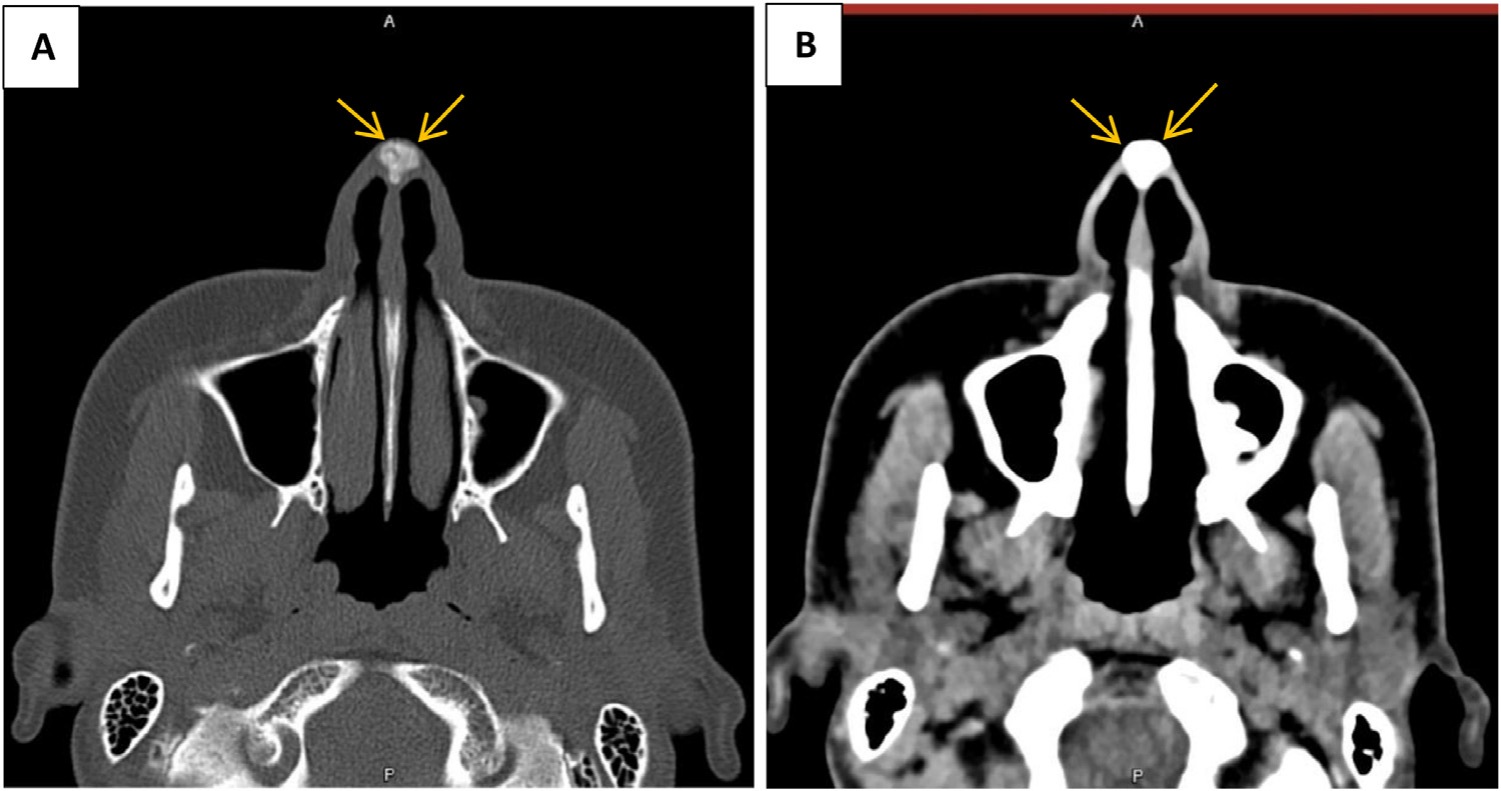
Axial CT scan bone window (A) and soft tissue window (B) shows a hyperdense calcified lesion at nasal tip (Arrows).

**Fig. 2:**
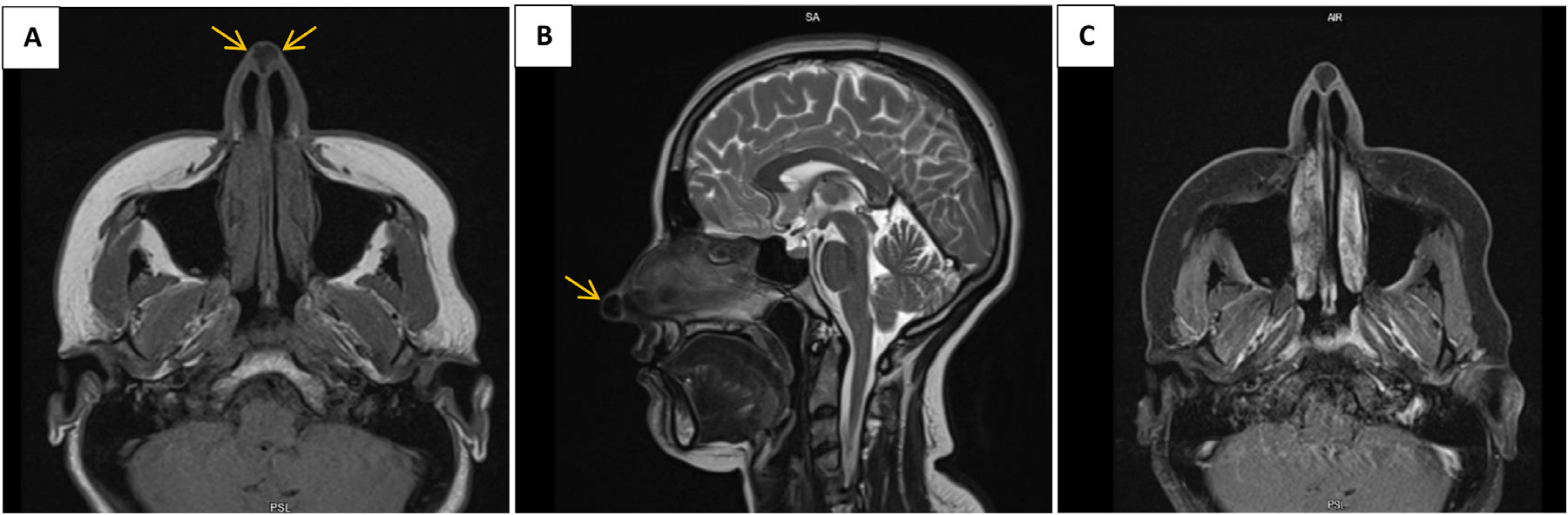
Axial T1 (A) and sagittal T2 (B) shows a midline low signal lesion at nasal tip (Arrows). Postcontrast axial T1 (C) shows only mild peripheral enhancement.

A 28-year-old woman with no significant medical history presented with a mass on the tip of her nose, which had been present for 3 years and had gradually enlarged over the past 3 months. On examination, there was a mass measuring approximately 1cm × 1cm with an elastic consistency. The overlying skin on the nose appeared thinned and telangiectatic. Ultrasound showed a solid, iso- to hypoechoic lesion without vascularity, measuring 11mm × 9mm in the nasal tip area. In a non-contrast CT scan of the face, a well-defined, homogeneous, hyperdense mass was observed at the tip of the nose (Fig. 1). MRI revealed a lesion that was hypointense on both T1 and T2 sequences, with a well-defined wall. On post-contrast images, only the wall of the mass showed enhancement (Fig. 2).

During surgery, similar to an open rhinoplasty approach, a nasal flap was elevated. A cystic mass with a well-defined wall was found between the lower lateral cartilages, adhered to the overlying skin. The mass was excised with its capsule intact. After performing tip-plasty, the nasal flap was repositioned and sutured.

**Fig. 3:**
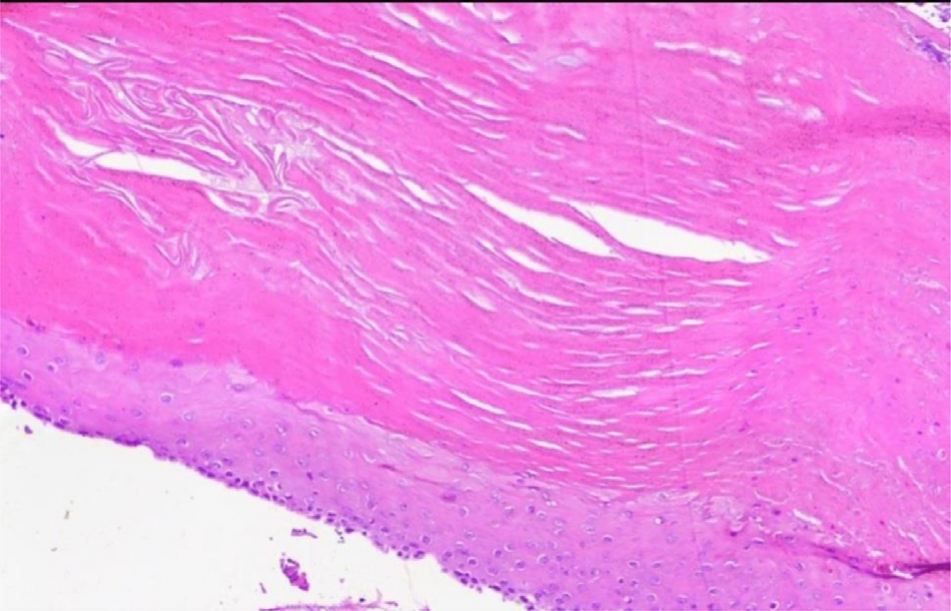
The photomicrograph displays a squamous epithelium lining that is stratified and does not have a granular layer. It has dense eosinophilic keratin, calcification, and a foreign body-type granulomatous reaction.

The macroscopic appearance revealed multiple fragments of creamy soft tissue. It was found that there was a pilar-shaped keratinous cyst, along with a multinucleated giant cell reaction and calcification (Fig. 3). Since this is a rare cause of nasal tip deformity, we decided to report it with the final diagnosis of keratinous cyst.

## Discussion

The external nose comprises bone and cartilage, which are enveloped by soft tissue. The structure comprises 2 nasal bones that connect with the nasal processes of the frontal bone and fuse laterally with the maxillae [3]. The nasal cartilages consist of an upper and lower pair. The upper lateral cartilages are connected to the bones, while the lower cartilages constitute the nasal tip and provide support to the columella [4].

CT and MR imaging protocols for evaluating cutaneous cancers and other nasal pathologies must encompass the tip of the nose, as it is often neglected by technologists. Radiologists must incorporate this region into their search pattern when analyzing cross-sectional studies of the brain, head, and neck. The field of view must encompass osseous and adjacent soft-tissue structures of the central skull base to ensure a comprehensive evaluation of the maxillary nerve course [5]. CT assessment may be conducted with or without iodinated contrast, while MR imaging can be executed with or without a gadolinium-based contrast agent. Typically, basic T1-weighted images, T2-weighted images with fat suppression, and postcontrast T1-weighted images with fat suppression are obtained. High-resolution steady-state free precession techniques, such as CISS and FIESTA sequences, enhance localization and tissue characterization [6].

The nose is among the most commonly affected sites of skin cancers, so it should be always considered when we face a pathology of the external apex nasi. This case report describes a 28-year-old woman with an atypical nasal tip mass, identified as a keratinous cyst exhibiting a multinucleated giant cell reaction and calcification. The mass, which had progressively increased over three years, was successfully excised via an open rhinoplasty technique. Preoperative imaging studies, such as ultrasound, noncontrast CT, and MRI, demonstrated distinctive characteristics of the mass, facilitating diagnosis and surgical planning.

The histopathological examination verified the existence of a pilar-shaped keratinous cyst, an uncommon occurrence at the nasal tip. A pillar-shaped keratinous cyst, or trichilemmal cyst, is often benign. These cysts arise from the external root sheath of hair follicles and are filled with keratin. They are predominantly located on the scalp and are often asymptomatic, hard, and mobile beneath the epidermis [7]. The multinucleated giant cell reaction and calcification highlighted the distinctiveness of this case. This report emphasizes the significance of including keratinous cysts in the differential diagnosis of nasal tip masses and highlights the necessity of thorough imaging and histopathological assessment for a conclusive diagnosis.

The findings enhance the existing literature on nasal tip deformities resulting from keratinous cysts, highlighting the necessity for increased awareness among clinicians and pathologists. Future research must concentrate on deepening the comprehension of the pathophysiology, clinical presentation, and optimal management strategies for these rare entities to improve patient outcomes. This case highlights the varied and occasionally unforeseen presentations of nasal masses, underscoring the necessity for comprehensive diagnostic and therapeutic strategies in their management.

## Patient consent

Written informed consent for the publication of this case report was obtained from the patient.
